# EMPOWERING STAFF TO MANAGE MEALTIME DIFFICULTIES AMONG RESIDENTS WITH DEMENTIA: A PILOT STUDY

**DOI:** 10.1093/geroni/igad104.2409

**Published:** 2023-12-21

**Authors:** Lígia Passos, João Tavares, Melissa Batchelor, Daniela Figueiredo

**Affiliations:** University of Aveiro, Aveiro, Aveiro, Portugal; University of Aveiro, Aveiro, Aveiro, Portugal; George Washington University, Washington, District of Columbia, United States; University of Aveiro, Aveiro, Aveiro, Portugal

## Abstract

People in advanced stages of dementia may experience mealtimes difficulties and depend on others for feeding. This pilot study aimed to evaluate the feasibility, acceptability, and preliminary effects of an educational intervention on mealtime difficulties of people with dementia, in a sample of 16 direct care workers and 9 people with dementia and was conducted in a nursing home. The sessions provided information about dementia, mealtime difficulties, and training on strategies and techniques. Feasibility was assessed through screening, eligibility, consent, retention, completion, and intervention adherence rates; and acceptability through a satisfaction questionnaire. The staff assessment included: burnout, job satisfaction, and knowledge about dementia and mealtime difficulties. In people with dementia, eating and feeding problems, and food intake were assessed. The main findings show that all feasibility rates, except for screening (89%), were excellent (100%). Direct care workers appreciated the intervention and perceived educational and practical benefits, although they reported needing more time to provide better assistance. Burnout levels did not change (mean 58.9 versus 58.6), but job satisfaction showed a positive trend (mean 71 versus 74.5) at the end of the intervention. Knowledge increased 37.5% compared to baseline. EdFED’s score presented slight improvement, while the oral intake of people with dementia increased after the intervention (91.1% versus 98.1%). The intervention was found to be feasible and acceptable. However, the educational intervention content needs improvement, and further studies with robust designs and larger samples are needed to better understand its effects on both direct care workers and people with dementia.

